# Severe nausea and vomiting in pregnancy: psychiatric and cognitive problems and brain structure in children

**DOI:** 10.1186/s12916-020-01701-y

**Published:** 2020-09-01

**Authors:** Hui Wang, Edmund T. Rolls, Xiujuan Du, Jingnan Du, Dexin Yang, Jiong Li, Fei Li, Wei Cheng, Jianfeng Feng

**Affiliations:** 1 Department of Developmental and Behavioral Pediatric & Child Primary Care/MOE-Shanghai Key Laboratory of Children’s Environmental Health, Xin Hua Hospital Affiliated to Shanghai Jiao Tong University School of Medicine, Shanghai, China; 2 Institute of Science and Technology for Brain-inspired intelligence, Fudan University, Shanghai, China; 3 Key Laboratory of Computational Neuroscience and Brain-Inspired Intelligence, Ministry of Education, Fudan University, Shanghai, China; 4 Department of Computer Science, University of Warwick, Coventry, CV4 7AL UK; 5 Oxford Centre for Computational Neuroscience, Oxford, OX1 4BH UK; 6 Department of Clinical Epidemiology, Aarhus University Hospital, Aarhus N, Denmark

**Keywords:** Nausea and vomiting, Cognitive performance, Psychiatric problems, Cortical structure, Cingulate cortex, Precuneus, Superior medial prefrontal cortex

## Abstract

**Background:**

Two studies have suggested that severe prolonged nausea and vomiting during pregnancy is associated with emotional and behavioral problems in offspring, with smaller sample size and short-term follow-up. Moreover, little information is available on the role of the brain structure in the associations.

**Methods:**

In a US-based cohort, the association was investigated between severe prolonged nausea and vomiting in pregnancy (extending after the second trimester and termed SNVP), psychiatric and cognitive problems, and brain morphology, from the Adolescent Brain Cognitive Development (ABCD) study, from 10,710 children aged 9–11 years. We validated the emotional including psychiatric findings using the Danish National Cohort Study with 2,092,897 participants.

**Results:**

SNVP was significantly associated with emotional and psychiatric problems (*t* = 8.89, Cohen’s *d* = 0.172, *p* = 6.9 × 10^−19^) and reduced global cognitive performance (*t* = − 4.34, *d* = − 0.085, *p* = 1.4 × 10^−5^) in children. SNVP was associated with low cortical area and volume, especially in the cingulate cortex, precuneus, and superior medial prefrontal cortex. These lower cortical areas and volumes significantly mediated the relation between SNVP and the psychiatric and cognitive problems in children. In the Danish National Cohort, severe nausea and vomiting in pregnancy were significantly associated with increased risks of behavioral and emotional disorders in children (hazard ratio, 1.24; 95% confidence interval, 1.16–1.33).

**Conclusions:**

SNVP is strongly associated with psychiatric and cognitive problems in children, with mediation by brain structure. These associations highlight the clinical importance and potential benefits of the treatment of SNVP, which could reduce the risk of psychiatric disorder in the next generation.

## Background

Nausea and vomiting during pregnancy (NVP), commonly known as morning sickness, affects as many as 80% of pregnant women [1]. Typical NVP occurs during the first trimester and is self-limiting, subsiding before 20 weeks of gestation [2]. It is considered to represent a healthy pregnancy, which has been linked to favorable obstetric and neonatal outcomes, including lower risk of miscarriage [3] and reduced odds for low birth weight and preterm birth [4, 5].

Clinically, around 15% of pregnant women continue to have NVP beyond 20 weeks of gestation [1]. Severe prolonged NVP correlates closely with vitamin B deficiency, chronic psychological stress, and weight loss [2]. These conditions play a crucial role in shaping fetal development and subsequent child brain and behavior development [6–9]. One study reported that severe prolonged NVP was associated with increased internalizing and externalizing behaviors in the offspring which reflect mood changes and conflict with others [10] and another reported decreased social responsiveness [11], highlighting the need for a large-scale evaluation of the long-term effects of exposure to severe prolonged NVP. As observed in two recent studies, clinical diagnosis of maternal hyperemesis gravidarum was associated with an increased risk of autism spectrum disorders in the offspring [12, 13]. Further, there is increasing evidence in general that exposure to adverse circumstances during pregnancy can significantly affect fetal brain development [14–17]. No previous study has examined the biological effects of severe prolonged NVP on brain morphology in children. Moreover, anatomical brain abnormalities play a crucial role in children with neurodevelopmental disorders [18, 19]. In this context, we performed a large-scale investigation of the association between severe prolonged nausea and vomiting in pregnancy and psychiatric and emotional problems, and brain structure, years later in children. We also assessed which brain differences may mediate the effects of severe prolonged NVP on psychiatric and cognitive problems, to potentially understand better what effects severe prolonged NVP may have.

We hypothesized that prenatal exposure to severe prolonged NVP is associated with poor cognitive performance and a high risk of emotional and psychiatric problems in children and that the underlying mechanisms could be related to structural differences in the brain. To test the hypothesis, the specific aims were as follows: (1) to assess the association between exposure to severe prolonged NVP and cognitive performance, emotional problems, and brain morphology in the children using data from the Adolescent Brain Cognitive Developmental (ABCD) Study [20]; (2) to examine whether brain morphology mediates the association between exposure to severe prolonged NVP and neurodevelopment in the children; and (3) to externally validate the results of severe nausea and vomiting (clinically diagnosed of hyperemesis gravidarum (HG) that requires antenatal hospital admission) and emotional and psychiatric problems, using data from the Danish National Registry System [21–27].

## Methods

### Participants

The dataset used for this investigation was from the Annual Curated Data Release 2.01 from the ABCD consortium (https://abcdstudy.org/). A total of 10,710 participants aged 9 to 11 years were included in the ABCD study, which was a large national-based longitudinal study that recruited children across 21 research sites across the USA [20]. The ABCD investigators obtained written and oral informed consent from parents and children, respectively [28]. More details of the study subjects and the collection are provided at the ABCD website (https://abcdstudy.org/scientists/protocols/) and also are described elsewhere [20, 29].

### Structural neuroimage processing

We obtained preprocessed structural magnetic resonance imaging (MRI) data (T1 and T2) using the ABCD pipeline, with all the data preprocessing procedures performed by the ABCD team as described in their image processing paper [29]. Briefly, the data preprocessing included the following procedures: (1) T1w and T2w structural images were corrected for gradient nonlinearity distortions; (2) T2w images were registered to T1w images; (3) intensity normalization and B1 inhomogeneity correction; (4) images were rigidly registered and resampled into alignment with a custom, in-house atlas created by the ABCD data preprocessing team for participants of this age; (5) FreeSurfer (version: v5.3.0) was used for cortical surface reconstruction and subcortical segmentation which included skull-stripping, white matter segmentation, initial mesh creation, correction of topological defects, generation of optimal white and pial surfaces, and nonlinear registration to a spherical surface-based atlas based on the alignment of sulcal/gyral patterns; (6) images were registered to a spherical atlas based on surface-based nonlinear registration, and the cerebral cortex was parcellated into 74 regions per hemisphere [30]; and (7) and finally, morphometric measures included the cortical area, volume, and thickness of each brain region and were used in our analysis.

The quality control of the processed images was done by the ABCD team. The trained staff manually reviewed the accuracy of cortical surface reconstruction, and only the data recommended for use was included in our analysis [29]. A total of 481 participants who failed to pass the quality control of the ABCD team were removed from the subsequent analysis, and there was no difference in the percentage of subjects who failed the quality control between the two groups. In addition, 360 participants who did not contain the full information of both the exposure to NVP and the structural images were excluded. Finally, 10,710 participants remained in this study, and the demographic characteristics of these participants are summarized in Table 1. All brain measurements and behavioral variables used in this study were collected at the ABCD baseline time when the average age was 119 months.

**Table 1:** Demographic characteristics of the 10,710 ABCD participants

	Controls (*N* = 9214)	SNVP (*N* = 1496)	Comparison	
			*t*/*χ*^2^ statistic	*p* value
Age (months)	119.06 ± 7.48	118.71 ± 7.37	1.704	0.088
Gender (male/female)	4358/4856	757/739	5.631	0.018
BMI	18.66 ± 4.08	19.48 ± 4.58	− 7.141	9.87 × 10^−13^
Parents income	7.70 ± 2.42	6.76 ± 2.95	13.48	4.47 × 10^−41^
Parents education	16.80 ± 2.65	15.55 ± 3.07	16.56	7.32 ×10^−61^
Puberty	1.58 ± 0.48	1.72 ± 0.55	− 9.935	3.71 × 10^−23^
Race (White/Black/Indian/others)	7151/1718/277/68	965/436/67/28	120.4	5.10 × 10^−28^
Use of tobacco (yes/no)	8062/1152	1260/236	11.47	7.09 × 10^−4^
Use of alcohol (yes/no)	6912/2302	1186/310	13.31	2.65 × 10^−4^
Use of marijuana (yes/no)	8767/447	1382/114	18.18	2.01 × 10^−5^
Use of cocaine/crack (yes/no)	9179/35	1485/11	1.556	0.212
Use of heroin/morphine (yes/no)	9204/10	1491/5	0.903	0.342
Use of oxytocin (yes/no)	9192/22	1489/7	0.470	0.493

The data was shown as mean ± standard deviation

**Table 2:** Characteristics of the Danish study population born between 1978 and 2012 at birth according to maternal hyperemesis gravidarum status (*N* = 2,092,897)

	Maternal hyperemesis gravidarum status	
	Yes (*N* = 21,282)	No (*N* = 2,071,615)
Characteristics	*N* (%)	*N* (%)
Sex		
Boys	9702 (45.6)	1,064,828 (51.4)
Girls	11,580 (54.4)	1,006,787 (48.6)
Preterm		
No	19,573 (92.0)	1,899,027 (91.7)
Yes	1088 (5.1)	95,375 (4.6)
Missing	621 (2.9)	77,213 (3.7)
Low birth weight		
No	20,146 (94.6)	1,961,425 (94.7)
Yes	840 (4.0)	80,443 (3.9)
Missing	296 (1.4)	29,747 (1.4)
Parity		
1	9488 (44.6)	922,209 (44.5)
2	7916 (37.2)	770,725 (37.2)
≥ 3	3878 (18.2)	378,681 (18.3)
Maternal age (years)		
≤ 25	7112 (33.4)	569,002 (27.5)
26–30	7619 (35.8)	785,543 (37.9)
31–35	4868 (22.9)	526,372 (25.4)
> 36	1683 (7.9)	190,698 (9.2)
Paternal age (years)		
≤ 25	3608 (17.0)	293,050 (14.1)
26–30	6683 (31.4)	659,191 (31.8)
31–35	5993 (28.2)	616,331 (29.8)
> 36	4393 (20.6)	413,205 (20.0)
Missing	605 (2.8)	89,838 (4.3)
Maternal education level		
0–9	7026 (33.0)	577,435 (27.9)
10–14	9257 (43.5)	905,428 (43.7)
≥ 15	4454 (20.9)	555,847 (26.8)
Missing	545 (2.6)	32,905 (1.6)
Maternal psychiatric disorders		
No	19,042 (89.5)	1,964,722 (94.8)
Yes	2240 (10.5)	106,893 (5.2)
Paternal psychiatric disorders		
No	19,991 (93.9)	1,981,118 (95.6)
Yes	1291 (6.1)	90,497 (4.4)
Maternal original		
Born in Denmark	16,103 (75.7)	1,853,108 (89.5)
Not born in Denmark	5151 (24.2)	213,826 (10.3)
Missing	28 (0.1)	4681 (0.2)
Maternal cohabitation status		
Yes	12,590 (59.2)	1,158,257 (55.9)
No	8688 (40.8)	911,630 (44.0)
Missing	4 (0.0)	1728 (0.1)

### Maternal nausea and vomiting during pregnancy

Information about NVP was collected through a structured maternal interview. Mothers in the ABCD study were asked if they had severe NVP extending past the 6th month of gestation, or NVP that was accompanied by weight loss, and were included in the group named SNVP studied here if they answered yes, and if the answer was no, they were in the control group (ABCD question dhx01, item devhx_10a3_p).

### Cognition measures

Cognitive development was assessed with NIH Toolbox (abcd_tbss01) which consists of seven validated and reliable psychometric tests (the Picture Vocabulary Test, the Flanker Inhibitory Control and Attention Test, the List Sorting Working Memory Test, the Dimensional Change Card Sort Test, the Pattern Comparison Processing Speed Test, the Picture Sequences Memory test, and the Oral Reading Recognition Test) [31, 32].

### Emotional and psychiatric problems

Emotional and behavioral problems were assessed by the Child Behavioral Checklist (CBCL), completed by the child’s caregiver. CBCL has high test-retest stability and good internal consistency and contains 113 items that measure broad scopes of child behavior across the past 6 months [33]. The resulting syndrome scores including anxious/depressed, withdrawn/depressed, somatic complaints, social problems, thought problems, rule-breaking behavior, and aggressive behavior were derived [34, 35]. Because this checklist includes some items related to psychiatric problems including depression that are associated with NVP (Additional file 1: Table S1), we refer to these measures in this paper as “emotional and psychiatric problems.”

More details of these behavior assessments are provided in Additional file 1: Table S2 [20, 28, 29, 31, 32, 36–39] and are also available at the ABCD website (https://abcdstudy.org/scientists-protocol.html).

### Statistical analysis

#### Association analysis

A linear mixed-effect model (LMM) was used to test the associations of the exposure to NVP with the brain morphometric measures and with the children’s cognitive scores from the NIH Cognitive Toolbox and the psychiatric problems scores from the Child Behavior Checklist noted above that are provided by ABCD. As recommended by the ABCD and used in many studies [40, 41], a linear mixed-effects model (LMM) was used to model the correlated observations within families due to twins and siblings and at sites. In this way, the LMM was specified to model family nested within the site. The LMM was implemented using the MATLAB function *fitlme*. A morphometry measurement or behavioral score was modeled as the dependent variable, and the exposure to SNVP and the nuisance covariates were modeled as fixed effects, while the family structures nested within sites were modeled as random effects. The following variables were used as nuisance covariates of no interest: children’s age, sex, body mass index, puberty score, race (coded as 3-column dummy variables), and parents’ income, number of years of education, use of tobacco, use of alcohol, use of marijuana, use of cocaine/crack, use of heroin/morphine, and use of oxytocin during pregnancy, in line with previous studies [40, 42, 43]. A *t*-statistic and Cohen’s *d* were calculated based on the coefficient of interest variable (the exposure to SNVP) in the linear mixed-effect model to reflect the association between the exposure to SNVP and the dependent variable. A positive *t* value means that the dependent variable is higher in the SNVP exposure group. Finally, false discovery rate (FDR) and Bonferroni corrections to correct the results for multiple comparisons were performed.

#### Mediation analysis

A standard mediation analysis was performed using the Mediation Toolbox developed by Tor Wager’s group (https://github.com/canlab/MediationToolbox), which has been widely used in neuroimaging studies [44, 45]. A standard 3-variable path model was used here [46], with the detailed methodology described in the supplementary material of [44]. Briefly, mediation analysis tests whether the covariance between two variables can be explained by the third variable (the mediator). The two hypotheses investigated the relation of cortical morphometry with psychiatric and cognitive problems and exposure to SNVP. For the first hypothesis, the independent (predictor) variable was the exposure to SNVP and the dependent (predicted) variable was the psychiatric problems total score. The proposed mediator (in the indirect path) was a morphometric measure. For the second hypothesis, the independent (predictor) variable was the exposure to SNVP and the dependent (predicted) variable was the Cognition Total Composite Score. The proposed mediator was a morphometric measure. Confounding variables as in the association analysis were regressed out in the mediation model. The significance of the mediation was estimated by the bias-corrected bootstrap approach (with 10,000 random samplings).

### External validation using the Danish nationwide cohort study

All the Danish data are obtained from national registers [22–27] for which detailed information is presented in Additional file 1: Table S3. All residents have a unique personal identification number that permits an accurate linkage of individual-level data from registers [21]. We conducted a population-based cohort study including all singleton live births born in Denmark during 1978–2012 (*N* = 2,100,158). We excluded 463 children who had errors in gestational age (< 154 or > 315 days) and 6798 children without links to their fathers. The final analyses included a total of 2,092,897 children (Additional file 1: Figure S1). We followed them from birth until the date of the first diagnosis of a psychiatric disorder, emigration, death, 18-year birthday, or 31 December 2016, whichever came first. From 1969 to 1993, the diagnostic system used was the Danish modification of the *International Classification of Disease, 8th revision (ICD-8)*, and from 1994 the *International Classification of Disease, 10th revision (ICD-10).*

Information on hyperemesis gravidarum (HG) was obtained from the Danish National Patient Register (DNPR). Diagnoses for HG are defined as *ICD-8* code: 638 and *ICD-10* code: O21 [47].

Clinical diagnoses for psychiatric disorders were identified from the DNPR and the Danish Psychiatric Central Research Register (DPCRR) [22, 23]. In order to focus on neuropsychiatric disorders with onset usually occurring in childhood and adolescence, and also to be consistent with previous publications based on Danish registers [48, 49], we categorized them into the following groups: pervasive developmental disorders including childhood autism; developmental disorders including language, learning, and motor skills disorders; and behavioral and emotional disorders with onset usually occurring in childhood and adolescence, including attentional-deficit/hyperactivity disorders, conduct disorders/oppositional defiant disorders, and emotional disorders. The diagnostic codes for *ICD-8* and *ICD-10* are provided in Additional file 1: Table S4*.* Based on previous studies using information from the Danish national registers, diagnoses for ADHD are defined according to *ICD-8* code: 30801 and *ICD-10* codes: F90 and F98.8. ADHD case was also defined when the individual had at least two redeemed prescriptions for ADHD-specific medication from the National Prescription Register [48]. The Anatomical Therapeutic Chemical codes for ADHD-specific medication were N06BA04 (methylphenidate) and N06BA09 (atomoxetine). Children who had redeemed N06BA07 (modafinil) were included as ADHD cases only if they had previously redeemed a prescription for either N06BA04 or N06BA09 [50].

The association of exposure to HG and behavioral and psychiatric problems in the offspring was estimated by the Cox proportional hazard model, with a child’s age as the time scale. Evaluation of log-minus-log survival curves showed that the curves were roughly parallel (Additional file 1: Figure S2). Covariates were identified a priori as potential confounding factors and/or risk factors for behavioral and emotional problems in children and adolescents. In the final model, the following potential confounders were included: sex of the child, calendar year of birth, maternal country of origin, maternal education and cohabitation status, parental age, and parental psychiatric disorder history before childbirth [48, 50]. To take account of the *ICD* code change, we restricted analyses to offspring born after 1994 when *ICD-10* codes were used.

#### Role of the funding source

The funding sources had no role in the study design; in the collection, analysis, and interpretation of the data; in the writing of the report; and in the decision to submit the paper for publication.

## Results

### Severe prolonged NVP is associated with psychiatric and cognitive problems in children aged 9–11 years

**Fig. 1:**
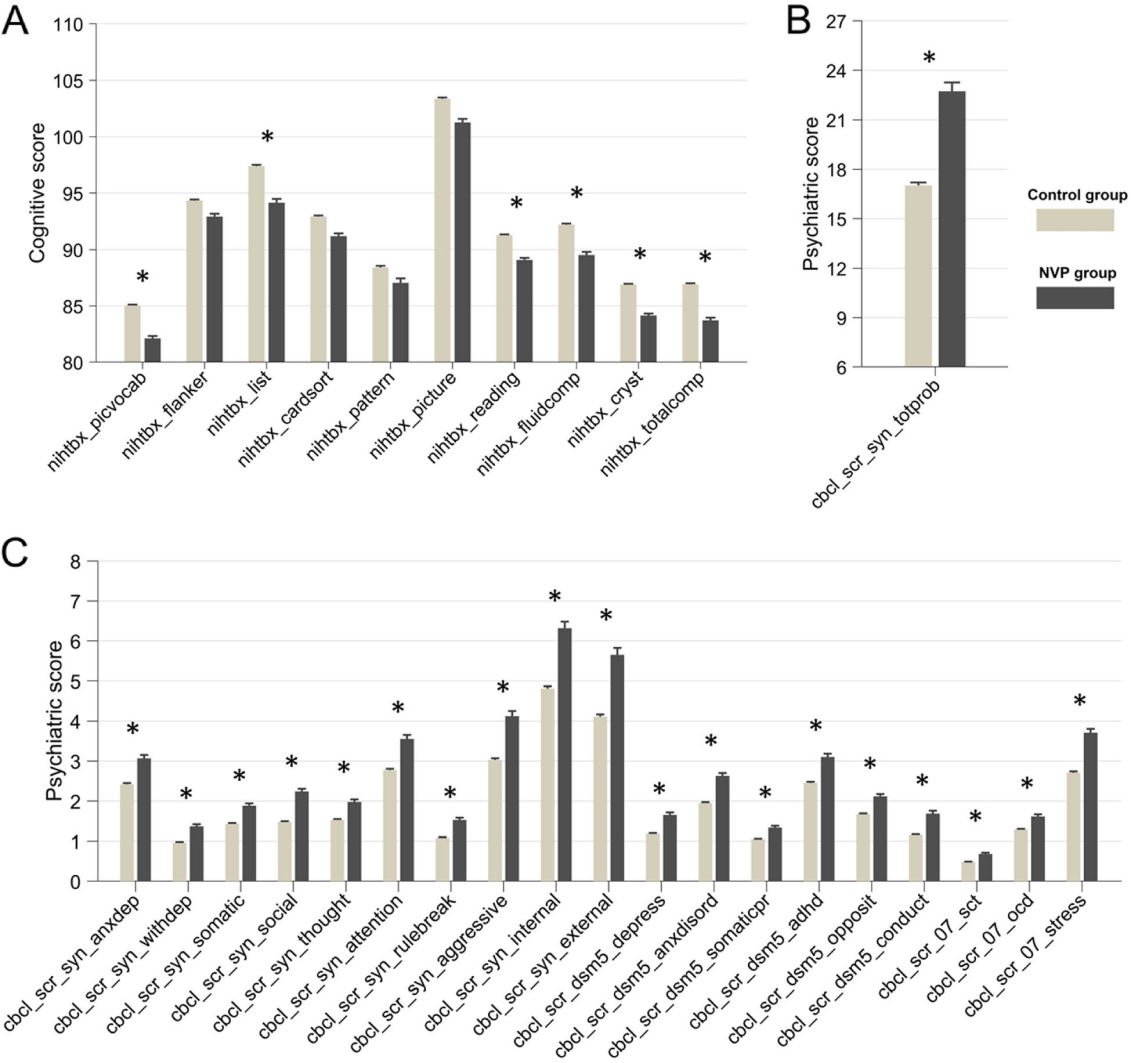
**a** A histogram showing the differences in cognitive measurements of 10,710 children between the children of mothers with prolonged nausea and vomiting in pregnancy (SNVP) and controls. The *Y* axis is the cognitive score (from the abcd_tbss) and the error bar is the SEM. All cognitive measurements except for flanker inhibitory control and attention (nihtbx_flanker), executive function (nihtbx_cardsort), language (nihtbx_picture), and pattern comparison processing speed (nihtbx_pattern) were significantly lower in the children with mothers with NVP during pregnancy (FDR corrected, *p* < 0.05). **b**, **c** Histograms showing the differences in psychiatric and emotional measures in the children of mothers with SNVP and controls. The *Y* axis is the psychiatric score. All psychiatric measurements were significantly lower in the group with exposure to SNVP during pregnancy (FDR corrected, *p* < 0.05). *FDR corrected *p* < 0.05. Note: nihtbx_picvocab: NIH Toolbox Picture Vocabulary Test Age 3+ v2.0 Uncorrected Standard Score; nihtbx_flanker: NIH Toolbox Flanker Inhibitory Control and Attention Test Ages 8-11 v2.0 Uncorrected Standard Score; nihtbx_list: NIH Toolbox List Sorting Working Memory Test Age 7+ v2.0 Uncorrected Standard Score; nihtbx_cardsort: NIH Toolbox Dimensional Change Card Sort Test Ages 8-11 v2.0 Uncorrected Standard Score; nihtbx_pattern: NIH Toolbox Pattern Comparison Processing Speed Test Age 7+ v2.0 Uncorrected Standard Score; nihtbx_picture: NIH Toolbox Picture Sequence Memory Test Age 8+ Form A v2.0 Uncorrected Standard Score; nihtbx_reading: NIH Toolbox Oral Reading Recognition Test Age 3+ v2.0 Uncorrected Standard Score; nihtbx_fluidcomp: Cognition Fluid Composite Uncorrected Standard Score; nihtbx_cryst: Crystallized Composite Uncorrected Standard Score; nihtbx_totalcomp: Cognition Total Composite Score Uncorrected Standard Score; cbcl_scr_syn_anxdep: Anxious/Depressed CBCL Syndrome Scale; cbcl_scr_syn_withdep: Withdrawn/Depressed CBCL Syndrome Scale; cbcl_scr_syn_somatic: Somatic Complaints CBCL Syndrome Scale; cbcl_scr_syn_social: Social Problems CBCL Syndrome Scale; cbcl_scr_syn_attention: Attention Problems CBCL Syndrome Scale; cbcl_scr_syn_rulebreak: Rule-Breaking Behavior CBCL Syndrome Scale; cbcl_scr_syn_aggressive: Aggressive Behavior CBCL Syndrome Scale; cbcl_scr_syn_internal: Internalizing Problems CBCL Syndrome Scale; cbcl_scr_syn_external: Externalizing Problems CBCL Syndrome Scale; cbcl_scr_dsm5_depress: Depressive Problems CBCL DSM5 Scale; cbcl_scr_dsm5_anxdisord: Anxiety Problems CBCL DSM5 Scale; cbcl_scr_dsm5_somaticpr: Somatic Problems CBCL DSM5 Scale; cbcl_scr_dsm5_adhd: ADHD CBCL DSM5 Scale; cbcl_scr_dsm5_opposit: Oppositional Defiant Problems CBCL DSM5 Scale; cbcl_scr_dsm5_conduct: Conduct Problems CBCL DSM5 Scale; cbcl_scr_07_sct: Sluggish Cognitive Tempo (SCT) CBCL Scale2007 Scale; cbcl_scr_07_ocd: Obsessive-Compulsive Problems (OCD) CBCL Scale2007 Scale; cbcl_scr_07_stress: Stress Problems CBCL Scale2007 Scale

All the psychiatric problems scores (abcd_cbcls01) were significantly higher in the group with SNVP with *t* values ranging from 5.10 to 9.09 (Bonferroni corrected all *p* < 1 × 10^−5^, Additional file 1: Table S1, Fig. 1b, c). The total psychiatric problems score was 22.7 in the SNVP group and 17.0 in the controls (*p* < 10^−10^, Additional file 1: Table S1), indicating that the children exposed to SNVP had a total psychiatric problems score that was 25.2% higher on average. The psychiatric problems associated with SNVP included ADHD, depression, and social problems (Additional file 1: Table S1).

Six out of ten cognitive scores (abcd_tbss01) were significantly lower in the group whose mother had severe prolonged nausea and vomiting during pregnancy (FDR correction, *p* < 0.05, Fig. 1a and Additional file 1: Table S1). The total cognitive performance score was 83.7 in the SNVP group and 86.9 in the controls (*p* = 1.4 × 10^−5^, Additional file 1: Table S1), indicating that the children exposed to SNVP had a total cognitive performance score that was 3.7% lower on average.

### Severe prolonged NVP is associated with low volume and area of some cortical regions

**Fig. 2:**
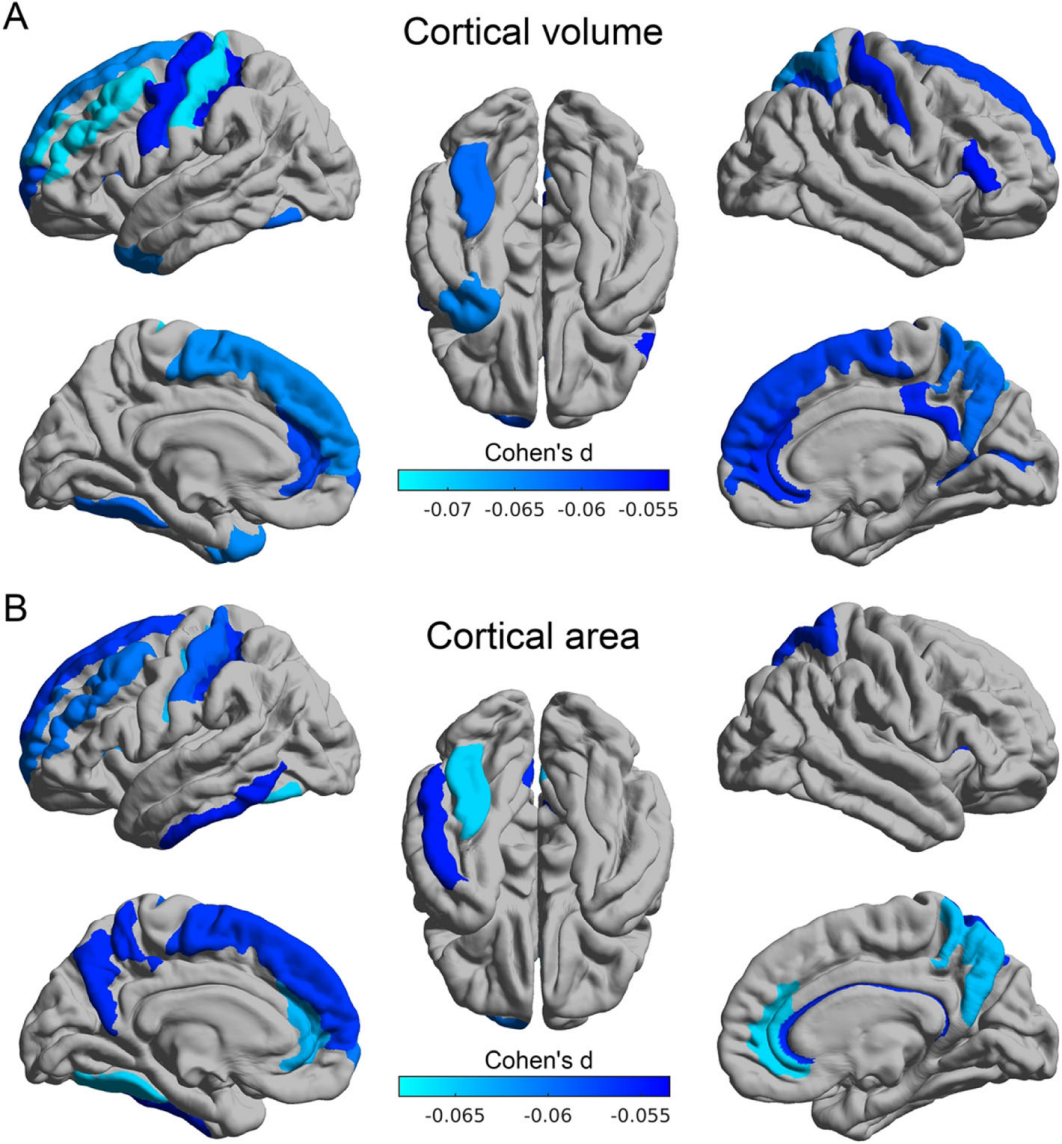
**a** Brain regions with their cortical volume significantly lower in children whose mothers had SNVP during pregnancy (FDR corrected, *p* < 0.05). **b** Brain regions with their cortical area significantly lower in the children whose mothers had SNVP during pregnancy (FDR corrected, *p* < 0.05)

The total cortical volume and area were significantly lower in the children with exposure to SNVP with *t* value − 4.02 (Cohen’s *d* = − 0.08, *p* = 6.0 × 10^−5^) and − 3.51 (*d* = − 0.07, *p* = 4.4 × 10^−4^) respectively. The reductions of volume were especially of the anterior and posterior cingulate cortex, precuneus, superior and middle frontal gyrus and superior medial prefrontal cortex, the dorsolateral prefrontal cortex, temporal pole, calcarine cortex, and post- and precentral gyrus (FDR corrected, *p* < 0.05, Fig. 2a, Additional file 1: Table S5). Similar brain areas, including the anterior and posterior cingulate cortex, precuneus, superior medial prefrontal cortex, inferior temporal cortex, insula, and postcentral cortex, showed significantly lower cortical areas in the children with exposure to NVP (FDR corrected, *p* < 0.05, Fig. 2b, Additional file 1: Table S5). No brain regions showed increased cortical volume and area in the SNVP group. No brain regions showed altered cortical thickness in the children with prenatal exposure to SNVP after FDR correction (*p* < 0.05).

### The lower cortical volume and area of some brain regions significantly mediate the association between exposure to SNVP and psychiatric and cognitive problems

**Fig. 3:**
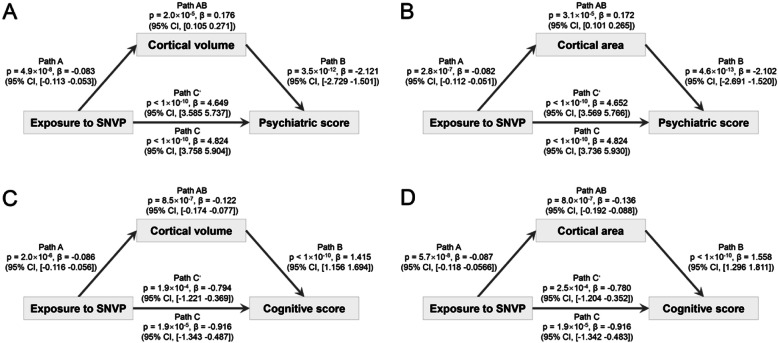
Mediation analysis of the effects of SNVP in the mothers on cognition and psychiatric problems that are mediated by brain structure. **a** The mediation by cortical volume from the exposure to SNVP on psychiatric problems was significant (*β* = 0.176, *p* = 2.0 × 10^−5^). Path A: the effect of the exposure to SNVP on the mediator (cortical volume). Path B: the effect of the mediator (cortical volume) on the outcome (psychiatric problems). Path C shows that the regression coefficient (beta value) of the exposure to SNVP on the cognition was high when the cortical volume was not taken into account. The beta values show the regression coefficient of the effect of the independent variable (the exposure to SNVP) on the outcome (psychiatric problems). Path C′ indicates the direct effect of the exposure to SNVP on the outcome (psychiatric problems) controlling for the mediator (cortical volume). Path C′ shows some reduction in the regression coefficient when the effect of the cortical volume was taken into account. Path AB indicates the extent to which taking the cortical volume into account can explain the 3.7% effect of the cortical area, which is significant as noted above at *p* = 2.0 × 10^−5^. **b** The mediation by cortical area from the exposure to SNVP on psychiatric problems was significant (*β* = 0.172, *p* = 3.1 × 10^−5^). **c** The mediation by cortical volume from the exposure to SNVP on cognition was significant (*β* = − 0.122, *p* = 8.5 × 10^−7^). **d** The mediation by cortical area from the exposure to SNVP on cognition was significant (*β* = − 0.136, *p* = 8.0 × 10^−7^)

The indirect effect of the exposure to SNVP on the psychiatric problems was significantly mediated by the mean cortical volume of the significant brain regions shown in Fig. 2a (3.7% of the total effect size measured by the variance explained (VE), *p* = 2.0 × 10^−5^, *β* = 0.176, 95% CI, 0.105 to 0.271, Fig. 3a) and by the mean cortical area of the significant brain regions shown in Fig. 2a (VE = 3.6%, *p* = 3.1 × 10^−5^, *β* = 0.172, 95% CI, 0.101 to 0.265, Fig. 3b). The mediation results for each brain area are shown in Additional file 1: Table S6, with the areas involved including the anterior cingulate cortex, superior and middle frontal gyrus, precuneus, and temporal pole (FDR correction, *p* < 0.05).

The indirect effect of the exposure to SNVP on cognitive performance was significantly mediated by the mean cortical volume of the significant brain regions shown in Fig. 2b (VE = 13.3%, *p* = 8.5 × 10^−7^, *β* = − 0.122, 95% CI, − 0.174 to − 0.077, Fig. 3c). The indirect effect of the exposure to SNVP on cognitive performance mediated by the mean cortical area of the significant brain regions shown in Fig. 2b was also significant (VE = 14.9%, *p* = 8.0 × 10^−7^, *β* = − 0.136, 95% CI, − 0.192 to − 0.088, Fig. 3d). The mediation results for each brain area are shown in Additional file 1: Table S7, with the areas involved including the anterior and posterior cingulate cortex, superior and middle frontal gyrus, precuneus, inferior temporal cortex, and insula (FDR correction, *p* < 0.05).

### External validation results using the Danish nationwide cohort study

In the Danish National Cohort, 21,282 (1.0%) children were born to mothers with hyperemesis gravidarum. The proportion of offspring born to mothers with HG increased over time (Additional file 1: Figure S3). Table 2 presents the baseline characteristics of the participants. Compared with unexposed offspring, exposed offspring were more likely to be born to younger mothers. Compared with mothers who did not experience HG, mothers with HG were more likely to have a lower level of education and more comorbid psychiatric disorders. Table 3 shows that compared to unexposed children, children of mothers with HG had increased risks of behavioral and emotional disorders (HR, 1.24; 95% CI, 1.16–1.33), attention-deficit/hyperactivity disorders (HR, 1.22; 95% CI, 1.12–1.33), conduct disorders/oppositional defiant disorders (HR, 1.31; 95% CI, 1.02–1.67), emotional disorders (HR, 1.40; 95% CI, 1.11–1.77), pervasive developmental disorders (HR, 1.18; 95% CI, 1.05–1.34), childhood autism (HR, 1.28; 95% CI, 1.06–1.56), and developmental disorders (HR, 1.36; 95% CI, 1.09–1.70). The results remained unchanged when restricting analyses to offspring born after 1994 (Additional file 1: Table S8).

**Table 3:** Incidence rate and hazard ratio of specific psychiatric disorders in offspring born during 1995–2012 in Denmark relating to maternal hyperemesis gravidarum

	No. of cases, *N*	Incidence rate, rate per 1000 person-years	Model 1, HR (95% CI)	Model 2, HR (95% CI)
Behavioral and emotional disorders*				
No HG	41,869	3.07	Ref	Ref
Maternal HG	595	3.67	1.27 (1.17–1.38)	1.20 (1.10–1.30)
Attention-deficit/hyperactivity disorders				
No HG	32,177	2.57	Ref	Ref
Maternal HG	437	2.85	1.23 (1.12–1.35)	1.16 (1.06–1.28)
Conduct disorders/oppositional defiant disorders				
No HG	2024	0.15	Ref	Ref
Maternal HG	29	0.18	1.31 (0.91–1.89)	1.06 (1.71–1.57)
Emotional disorders				
No HG	2884	0.21	Ref	Ref
Maternal HG	44	0.27	1.34 (1.00–1.81)	1.33 (0.98–1.89)
Pervasive developmental disorders				
No HG	17,478	1.31		
Maternal HG	232	1.46	1.23 (1.08–1.40)	1.19 (1.05–1.36)
Childhood autism				
No HG	6877	0.50	Ref	Ref
Maternal HG	100	0.61	1.32 (1.08–1.61)	1.19 (0.97–1.45)
Developmental disorders^#^				
No HG	3362	0.24	Ref	Ref
Maternal HG	55	0.33	1.43 (1.10–1.86)	1.33 (1.02–1.75)

*HG* hyperemesis gravidarum, *HR* Hazard ratio, *Model 1* children’s age at time scale, *Model 2* children’s age at time scale, sex, year of birth, parity, parental age at birth, maternal education level, maternal country of origin, maternal cohabitation, and parental psychiatry disorders

*Behavioral and emotional disorders with onset usually occurring in childhood and adolescence

^#^Developmental disorders including language, learning, and motor skills disorders

## Discussion

In this large, nationally representative cohort of 10,710 US children, our findings provide the first evidence that prenatal exposure to prolonged severe nausea and vomiting in pregnancy (SNVP) is associated with a range of psychiatric problems including ADHD symptoms, and with poor cognitive performance. External validation from the Danish National Cohort also suggested that severe nausea and vomiting in pregnancy is associated with psychiatric problems (including ADHD), and behavioral and emotional problems. The findings also show that children with prenatal exposure to SNVP have significantly reduced total cortical volume and area; and that the particular brain areas with significant reductions include the anterior and posterior cingulate cortex, the precuneus, the superior medial prefrontal cortex, the superior and middle frontal gyri, the temporal lobe, the insula, the calcarine cortex, and the postcentral gyrus. Further mediation analyses showed that the reduced volume or area of many of these brain regions significantly mediates the effects of SNVP on psychiatric problems and on cognition. These findings provide a unique contribution demonstrating that prenatal exposure to prolonged and severe NVP is associated with psychiatric problems and poor cognitive performance in the offspring and that the effects are mediated via the altered brain morphology. The findings highlight the clinical importance of attention to prolonged nausea and vomiting in pregnant women for mental health in the offspring.

Previous studies on the association of exposure to severe prolonged NVP and the risk of adverse health outcomes in the offspring are scarce. Two are mentioned in the introduction [10, 11], and these much smaller studies (*n* = 560 and 287, respectively) reported some changes in cognition and emotional and behavioral problems. The present study was much more powerful than previous studies, with a very large sample (*n* = 10,710) demonstrating that a wide range of psychiatric (Table S2) and cognitive measures (Table S2) are associated with severe prolonged NVP and with external-validation of the major psychiatric findings in an independent national sample (*n* = 2,092,897, Table 3).

No previous study has examined the association between exposure to severe prolonged NVP and brain morphology in the offspring. We found that exposure to SNVP is associated with reduced cortical areas to the entire brain surface. Studies in humans and primates have indicated that early brain development is characterized by an initial expansion of the cortical surface areas [51–53]. During the early stage of development, the expansion of the cortical areas may be particularly susceptible to environmental insults [54]. Accumulating evidence suggested that fetal exposure to adverse intrauterine exposures, i.e., cocaine, alcohol, cigarettes, or air pollution, is associated with reduced cortical areas [55–59]. For example, two studies have reported that fetal exposure to maternal psychosocial stress (depression/anxiety) during the second trimester of pregnancy is associated with decreased cortical thickness/volume [17, 60].

The brain areas that we have found to be significantly related to SNVP include anterior and posterior cingulate gyrus, precuneus, temporal pole, inferior temporal cortex, prefrontal areas including the superior and middle frontal gyri, and the superior medial prefrontal cortex, and pre- and postcentral gyri. The anterior cingulate cortex is involved in emotion and relating actions to rewards and punishers and is implicated in psychiatric disorders including ADHD and depression [61–63]. A previous case-control study demonstrated that individuals with ADHD had a smaller total cortical volume (7.3%), and surface area (4.3%), compared to controls, and the reduced surface area in the precuneus may be a major driver of the volume differences [64]. In the present investigation, the mediation analyses showed that reduced cortical area in the precuneus partly mediated the association of exposure to SNVP and emotional and psychiatric problems in offspring. The precuneus is a notable region for its role in awareness, episodic memory, and visuospatial processing, suggesting the potential relevance of this region for ADHD-related symptoms [65], for autism [66] and depression [67], and the precuneus is closely connected with the posterior cingulate cortex [61]. Prefrontal cortical areas are also linked to ADHD [68]. Our results from the Danish national population study confirmed that maternal HG is associated with a 44% increased risk of ADHD in the offspring.

These brain areas with regions significantly related to SNVP have also been consistently linked with cognitive functions [59, 69]. The superior and middle frontal gyri are involved in cognitive execution networks, such as reasoning, planning, and language comprehension [59]. The superior medial prefrontal cortex and frontal pole are also involved in cognitive functions, including planning [70]. Therefore, the reductions in these brain areas may help to account for the observed significant associations between SNVP and lower cognitive performance in the offspring.

There are many changes in the brain structure during development, including in the area, volume, and thickness of different cortical areas, and such differences are found in disorders like attention-deficit/hyperactivity disorder and autism spectrum disorder and in other childhood adversities [71–73]. Differences in these measures may reflect, for example, the extent of neuronal dendrites in a cortical area, and the number of synapses on each neuron. Studies with large populations are needed for reliable results. A key feature of the current investigation is that the morphometric effects were measured in 10,710 children, a study sample larger than that in most previous studies [73]. In terms of the interpretation of the reduced volumes and areas reported here associated with SNVP, we have reported in another study of associations involving maternal age at childbirth of the 10,710 participants, in which there was a linear relationship between the reduction in total cortical volume and area in children, and their behavioral and cognitive problems of the type described here [74].

### Possible mechanisms

The exact mechanism underlying the association between prenatal exposure to prolonged severe NVP and neurodevelopmental problems, including differences in the brain structure and psychiatric and cognitive problems, are not closely understood. First, it is plausible that altered brain morphology may be a possible mechanism via which exposure to severe prolonged NVP is associated with increased psychiatric, emotional, and cognitive problems. Second, severe prolonged NVP may indicate an environmental insult during pregnancy since severe prolonged NVP correlates closely with greater maternal stress [75]. Maternal elevated levels of stress during pregnancy could increase the release of placental corticotrophin-releasing hormone (pCRH), which could be produced from the placenta as early as the eighth week of gestation and increase exponentially across the gestation stage [76]. Notably, pCRH synthesis increase is responsive to maternal stress signals [76]. pCRH represents an integrative pathway through which diverse prenatal environmental insults inform the fetus of the condition and shape the fetal developmental trajectories [77]. Considerable evidence indicates that fetal exposure to excessive pCRH concentrations is associated with behavioral consequences during infancy and neuropsychiatric outcomes in childhood [78, 79]. Another plausible interpretation is that maternal stress induced by HG could result in elevated maternal cortisol levels during pregnancy [12]. Because cortisol can pass through the immature blood-brain barrier and targets glucocorticoid receptors, fetal exposure to elevated cortisol is associated with increased concentrations of glucocorticoids, which may play a critical role in modulating normal brain development [80, 81]. This hypothesis is also supported by animal models demonstrating that exposure to prenatal maternal stress is associated with changes in brain morphometry [82].

### Strengths and limitations

Our study had several strengths. First, the very large sample sizes facilitate generalization of the findings to other populations. Second, because comprehensive data is available in the ABCD study, we were able to control for a variety of potential confounding factors. Third, the participants in the current study were preadolescent children, so the morphological findings were unlikely to be confounded by smoking or substance use. The results described show that there is an important association between prolonged severe nausea and vomiting in pregnancy and psychiatric and cognitive problems in children, which are of clinical relevance, suggesting that interventions to limit the prolonged severe nausea and vomiting in pregnancy may be important.

Our study also has some limitations. First, maternal NVP was obtained through self-reports which might be subject to measurement error as in any observational study; however, if present, any misclassification should be non-differential with respect to the outcome measures and would thus have underestimated the true association. Additionally, the prevalence of prolonged severe NVP in our study is 13.98%; this is comparable to the prevalence of 10–15% reported in the general population. Nevertheless, in the external validation study, SNVP was ascertained according to clinical diagnosis, and the findings were similar. Second, we do not have treatment data in the ABCD dataset for SNVP. Treatment of NVP may be a marker of greater severity [83], which may magnify the strength of the association. Third, the use of tobacco, alcohol, and marijuana was higher in the SNVP group, and although we controlled for these factors in all analyses, the exposed and unexposed group may differ in some characteristics that were not measured, and thus, residual confounding cannot be ruled out. Fourth, our findings are preliminary. In the current analysis, cognitive performance, behavioral and emotional problems, and brain morphology were measured concurrently. Further studies with assessment at multiple time points to track neuropsychiatric development are warranted to clarify how these relationships change over time.

In Denmark, the comprehensive administrative databases allowed us to access maternal hyperemesis gravidarum that was obtained in an objective manner, which could reduce the chance of recall bias [26]. Moreover, the Danish registers have diagnostic information on mental disorders for the whole population, and all treatments will be provided through the government health care system free of charge to all residents [84]. Nevertheless, the register-based study could not capture individuals who do not seek treatment [23]. Although we adjusted for a number of potential confounders, we did not have information on several other factors such as maternal alcohol consumption. Therefore, the strengths and weaknesses of the Danish study contrast with and complement those of the ABCD cohort. Furthermore, even if these two cohorts have different, unrelated sources of bias, the results are comparable, suggesting that the associations observed in this study are unlikely to be explained by unmeasured confounders [85].

## Conclusions

Our results show for the first time that prolonged severe NVP is associated with robust differences in the brain structure which mediate psychiatric and emotional problems and reduced cognitive performance in the offspring during childhood. The findings highlight the importance of attention to pregnant women with severe prolonged NVP, of the need for investigations of whether interventions to limit severe prolonged NVP are beneficial, and of the relevance in considering the prenatal exposure to severe prolonged NVP as a risk factor in the clinical assessment of children with mental health disorders. The fact that these brain differences and the associated psychiatric and cognitive problems were present as long after birth as 9–11 years is an indication that these differences may be relevant through to adulthood. These findings highlight the clinical importance and potential benefits of treatment for prolonged nausea and vomiting in pregnant women, and early screening of the offspring, which could help to reduce the risk of psychiatric disorder in the next generation.

## Supplementary information

**Additional file 1: Table S1.** Detailed description of participants in the ABCD study. **Table S2.** The difference in cognitive and psychiatric measurements between the exposure to SNVP during pregnancy and control groups. **Table S3.** Brain regions with their cortical volume or area significantly altered in the children whose mothers had SNVP during pregnancy (FDR corrected, *p*<0.05). **Table S4.** The mediations on psychiatric problems implemented in the children by the volume and area of different cortical regions in the effects of exposure to severe and prolonged nausea and vomiting in pregnancy (FDR corrected, p<0.05). **Table S5.** The mediations implemented in the children by the volume and area of different cortical regions in the effects of exposure to SNVP on cognition (FDR corrected, p<0.05). **Table S6.** Detailed description of registers used in the study. **Table S7.** The diagnostic classification of psychiatric disorders according to ICD-8 and ICD-10 system. **Table S8.** Incidence rate and hazard ratio of specific psychiatric disorders in offspring born during 1995-2012 in Denmark according to maternal hyperemesis gravidarum. **Figure S1.** Flowchart showing the identification of the eligible participants and analysis sample. **Figure S2.** The log-minus-log survival curve. **Figure S3.** The proportion of offspring born to mothers with hyperemesis gravidarum by birth year.

## Data Availability

The datasets used and/or analyzed during the current study are available from the corresponding author on reasonable request.

## Abbreviations

NVP: Nausea and vomiting during pregnancy
ABCD: Adolescent Brain Cognitive Developmental
HG: Hyperemesis gravidarum
CBCL: Child Behavioral Checklist
ADHD: Attention-deficit/hyperactivity disorders
