# Supplementary material for: Severe nausea and vomiting in pregnancy: psychiatric and cognitive problems and brain structure in children
Source: BMC Med. 2020 Sep 1;18:228. doi: 10.1186/s12916-020-01701-y (PMC7460800; doi:10.1186/s12916-020-01701-y)
Supplement: Supplementary file 1 — Additional file 1: Table S1. Detailed description of participants in the ABCD study. Table S2. The difference in cognitive and psychiatric measurements between the exposure to SNVP during pregnancy and control groups. Table S3. Brain regions with their cortical volume or area significantly altered in the children whose mothers had SNVP during pregnancy (FDR corrected, p<0.05). Table S4. The mediations on psychiatric problems implemented in the children by the volume and area of different cortical regions in the effects of exposure to severe and prolonged nausea and vomiting in pregnancy (FDR corrected, p<0.05). Table S5. The mediations implemented in the children by the volume and area of different cortical regions in the effects of exposure to SNVP on cognition (FDR corrected, p<0.05). Table S6. Detailed description of registers used in the study. Table S7. The diagnostic classification of psychiatric disorders according to ICD-8 and ICD-10 system. Table S8. Incidence rate and hazard ratio of specific psychiatric disorders in offspring born during 1995-2012 in Denmark according to maternal hyperemesis gravidarum. Figure S1. Flowchart showing the identification of the eligible participants and analysis sample. Figure S2. The log-minus-log survival curve. Figure S3. The proportion of offspring born to mothers with hyperemesis gravidarum by birth year. [file 12916_2020_1701_MOESM1_ESM.docx]

**Supplementary Material**

**Severe nausea and vomiting in pregnancy: psychiatric and cognitive problems, and brain structure in children**

Hui Wang^1,#^, Edmund T. Rolls^2,4,5,#,*^, Xiujuan Du^1^, Jingnan Du^2^, Dexin Yang^2^, Jiong Li^1,6^,

Fei Li^1,*^, Wei Cheng^2, 3,*^ and Jianfeng Feng^2,3,4^

1. Department of Developmental and Behavioral Pediatric & Child Primary Care/MOE-Shanghai Key Laboratory of Children’s Environmental Health, Xin Hua Hospital Affiliated to Shanghai Jiao Tong University School of Medicine, Shanghai, China

2. Institute of Science and Technology for Brain-inspired intelligence, Fudan University, Shanghai, China

3. Key Laboratory of Computational Neuroscience and Brain-Inspired Intelligence (Fudan University), Ministry of Education, 200433, China

4. Department of Computer Science, University of Warwick, Coventry CV4 7AL, UK

5. Oxford Centre for Computational Neuroscience, Oxford OX1 4BH, UK

6. Department of Clinical Epidemiology, Aarhus University Hospital, Aarhus N, Denmark

***Authors for correspondence**

Dr. Wei Cheng, Institute of Science and Technology for Brain-inspired Intelligence, Shanghai, 200433, China, E-mail address: wcheng@fudan.edu.cn

Prof. Fei Li, Department of Developmental and Behavioral Pediatric&Child Primary Care, Xinhua Hospital Affiliated To Shang Jiaotong University School of Medicine, Shanghai.

Email: feili@shsmu.edu.cn.

# These two authors contributed equally to the manuscript and considered as co-first authors.

*These two authors are considered as co-corresponding authors.

**Table S1. The difference in cognitive and psychiatric measurements between the exposure to** **SNVP during pregnancy and** **control groups.**

| **NIH Toolbox Cognition Battery** | | | | | | | | | | | |
| --- | --- | --- | --- | --- | --- | --- | --- | --- | --- | --- | --- |
| **Cognitive measurement*** | **Controls** | **SNVP** | **t value** | **Cohen's d** | **p value** | **Cognitive measurement** | **Controls** | **SNVP** | **t value** | **Cohen's d** | **p value** |
| nihtbx_picvocab | 85.0$\pm$8.07 | 82.1$\pm$7.91 | -4.37 | -0.085 | 1.3×10^-5^ | nihtbx_picture | 103.3$\pm$12.1 | 101.3$\pm$12.0 | -1.74 | -0.034 | 0.081 |
| nihtbx_flanker | 94.3$\pm$8.86 | 92.9$\pm$9.80 | -1.44 | -0.028 | 0.151 | nihtbx_reading | 91.3$\pm$6.80 | 89.1$\pm$6.93 | -4.59 | -0.090 | 4.4×10^-6^ |
| nihtbx_list | 97.4$\pm$11.8 | 94.1$\pm$12.6 | -3.30 | -0.064 | 9.5×10^-4^ | nihtbx_fluidcomp | 92.2$\pm$10.4 | 89.5$\pm$10.9 | -2.54 | -0.050 | 0.011 |
| nihtbx_cardsort | 92.9$\pm$9.36 | 91.2$\pm$9.63 | -1.81 | -0.035 | 0.071 | nihtbx_cryst | 86.9$\pm$6.98 | 84.1$\pm$6.88 | -5.39 | -0.105 | 7.2×10^-8^ |
| nihtbx_pattern | 88.4$\pm$14.5 | 87.1$\pm$15.0 | -0.75 | -0.015 | 0.455 | nihtbx_totalcomp | 86.9$\pm$8.94 | 83.7$\pm$9.12 | -4.34 | -0.085 | 1.4×10^-5^ |
| **Parent Child Behavior Checklist Raw Scores Aseba** | | | | | | | | | | | |
| **Psychiatric measurement**** | **Controls** | **SNVP** | **t value** | **Cohen's d** | **p value** | **Psychiatric measurement** | **Controls** | **SNVP** | **t value** | **Cohen's d** | **p value** |
| cbcl_scr_syn_anxdep | 2.42$\pm$3.00 | 3.07$\pm$3.31 | 7.30 | 0.141 | 3.0×10^-13^ | cbcl_scr_dsm5_depress | 1.18$\pm$1.91 | 1.65$\pm$2.41 | 6.96 | 0.135 | 3.6×10^-12^ |
| cbcl_scr_syn_withdep | 0.96$\pm$1.62 | 1.37$\pm$2.06 | 6.26 | 0.121 | 3.9×10^-10^ | cbcl_scr_dsm5_anxdisord | 1.95$\pm$2.36 | 2.63$\pm$2.69 | 9.09 | 0.176 | 1.1×10^-19^ |
| cbcl_scr_syn_somatic | 1.43$\pm$1.88 | 1.88$\pm$2.24 | 7.06 | 0.137 | 1.8×10^-12^ | cbcl_scr_dsm5_somaticpr | 1.05$\pm$1.47 | 1.34$\pm$1.68 | 5.87 | 0.114 | 4.4×10^-9^ |
| cbcl_scr_syn_social | 1.47$\pm$2.15 | 2.24$\pm$2.63 | 8.74 | 0.169 | 2.8×10^-18^ | cbcl_scr_dsm5_adhd | 2.45$\pm$2.86 | 3.10$\pm$3.21 | 6.20 | 0.120 | 6.0×10^-10^ |
| cbcl_scr_syn_thought | 1.53$\pm$2.10 | 1.98$\pm$2.50 | 6.69 | 0.129 | 2.3×10^-11^ | cbcl_scr_dsm5_opposit | 1.67$\pm$1.97 | 2.12$\pm$2.20 | 6.44 | 0.125 | 1.2×10^-10^ |
| cbcl_scr_syn_attention | 2.77$\pm$3.34 | 3.55$\pm$3.85 | 6.54 | 0.127 | 6.4×10^-14^ | cbcl_scr_dsm5_conduct | 1.15$\pm$2.17 | 1.69$\pm$2.79 | 5.10 | 0.099 | 3.5×10^-7^ |
| cbcl_scr_syn_rulebreak | 1.08$\pm$1.72 | 1.53$\pm$2.18 | 5.34 | 0.103 | 9.5×10^-8^ | cbcl_scr_07_sct | 0.48$\pm$0.94 | 0.68$\pm$1.22 | 5.85 | 0.113 | 5.0×10^-9^ |
| cbcl_scr_syn_aggressive | 3.03$\pm$4.10 | 4.12$\pm$4.96 | 6.87 | 0.133 | 6.8×10^-12^ | cbcl_scr_07_ocd | 1.29$\pm$1.77 | 1.61$\pm$2.03 | 6.37 | 0.123 | 1.9×10^-15^ |
| cbcl_scr_syn_internal | 4.81$\pm$5.33 | 6.32$\pm$6.32 | 8.37 | 0.162 | 6.2×10^-17^ | cbcl_scr_07_stress | 2.71$\pm$3.19 | 3.71$\pm$3.83 | 8.70 | 0.168 | 3.9×10^-18^ |
| cbcl_scr_syn_external | 4.11$\pm$5.48 | 5.65$\pm$6.75 | 6.78 | 0.131 | 1.2×10^-11^ | cbcl_scr_syn_totprob | 17.02$\pm$16.9 | 22.74$\pm$20.8 | 8.89 | 0.172 | 6.9×10^-19^ |

**Note:** *A high cognitive score means better performance, and a high psychiatric score means a worse mental state. All cognitive measures were lower in the group with the exposure to SNVP.

**All psychiatric measures were higher in the group with the exposure to SNVP, indicating that the children with exposure to SNVP tend to have psychiatric problems.

**nihtbx_picvocab**: NIH Toolbox Picture Vocabulary Test Age 3+ v2.0 Uncorrected Standard Score; **nihtbx_flanker**: NIH Toolbox Flanker Inhibitory Control and Attention Test Ages 8-11 v2.0 Uncorrected Standard Score; **nihtbx_list**: NIH Toolbox List Sorting Working Memory Test Age 7+ v2.0 Uncorrected Standard Score; **nihtbx_cardsort**: NIH Toolbox Dimensional Change Card Sort Test Ages 8-11 v2.0 Uncorrected Standard Score; **nihtbx_pattern**: NIH Toolbox Pattern Comparison Processing Speed Test Age 7+ v2.0 Uncorrected Standard Score; **nihtbx_picture**: NIH Toolbox Picture Sequence Memory Test Age 8+ Form A v2.0 Uncorrected Standard Score; **nihtbx_reading**: NIH Toolbox Oral Reading Recognition Test Age 3+ v2.0 Uncorrected Standard Score; **nihtbx_fluidcomp**: Cognition Fluid Composite Uncorrected Standard Score; **nihtbx_cryst**: Crystallized Composite Uncorrected Standard Score; **nihtbx_totalcomp**: Cognition Total Composite Score Uncorrected Standard Score; **cbcl_scr_syn_anxdep**: Anxious/Depressed CBCL Syndrome Scale; **cbcl_scr_syn_withdep**: Withdrawn/Depressed CBCL Syndrome Scale; **cbcl_scr_syn_somatic**: Somatic Complaints CBCL Syndrome Scale; **cbcl_scr_syn_social**: Social Problems CBCL Syndrome Scale; **cbcl_scr_syn_attention**: Attention Problems CBCL Syndrome Scale; **cbcl_scr_syn_rulebreak**: Rule-Breaking Behavior CBCL Syndrome Scale; **cbcl_scr_syn_aggressive**: Aggressive Behavior CBCL Syndrome Scale; **cbcl_scr_syn_internal**: Internalizing Problems CBCL Syndrome Scale; **cbcl_scr_syn_external**: Externalizing Problems CBCL Syndrome Scale; **cbcl_scr_dsm5_depress**: Depressive Problems CBCL DSM5 Scale; **cbcl_scr_dsm5_anxdisord**: Anxiety Problems CBCL DSM5 Scale; **cbcl_scr_dsm5_somaticpr**: Somatic Problems CBCL DSM5 Scale; **cbcl_scr_dsm5_adhd**: ADHD CBCL DSM5 Scale; **cbcl_scr_dsm5_opposit**: Oppositional Defiant Problems CBCL DSM5 Scale; **cbcl_scr_dsm5_conduct**: Conduct Problems CBCL DSM5 Scale; **cbcl_scr_07_sct**: Sluggish Cognitive Tempo (SCT) CBCL Scale2007 Scale; **cbcl_scr_07_ocd**: Obsessive-Compulsive Problems (OCD) CBCL Scale2007 Scale; **cbcl_scr_07_stress**: Stress Problems CBCL Scale2007 Scale.

Table S2 Detailed description of participants and measurements in the ABCD study

| **Participants** | |
| --- | --- |
|  | The dataset used for this investigation was from the Annual Curated Data Release 2.0 from the ABCD consortium (<https://abcdstudy.org/>). A sample of 10,713 participants aged 9 to 11 years was included from the ABCD study, which is a large national-based longitudinal study that recruited children across 21 research sites across the US. At each ABCD data collection site, participants were predominantly recruited through local elementary and charter schools. ABCD employed a probability sampling strategy to identify schools within the 21 catchment areas as the primary method for contacting and recruiting eligible children and their parents. The ABCD investigators obtained written and oral informed consent from parents and children, respectively. The participants in the ABCD study closely match the US population of 9 to 11 years old children on several key demographic variables, including gender, race/ethnicity, household income, and parental education and marital status. More details of the subjects, and the collection and preprocessing parameters of the data are provided at the ABCD website (https://abcdstudy.org/scientists/protocols/) and also are described elsewhere. |
| **Cognition measures** | |
|  | Cognitive abilities were assessed by the ABCD *Youth NIH TB Summary Scores* (abcd_tbss01) which consists of 10 validated and reliable psychometric test scores: Picture Vocabulary Test Score (nihtbx_picvocab); Flanker Inhibitory Control and Attention Test Score (nihtbx_flanker); List Sorting Working Memory Score (nihtbx_list); Dimensional Change Card Sort Test Score (nihtbx_cardsort); Pattern Comparison Processing Speed Test Score (nihtbx_pattern); Picture Sequence Memory Test Score (nihtbx_picture); Oral Reading Recognition Test Score (nihtbx_reading); Cognition Fluid Composite Score (nihtbx_fluidcomp); Crystallized Composite Score (nihtbx_cryst); Cognition Total Composite Score (nihtbx_totalcomp). A high score means better cognitive ability. |
| **Emotional and** **psychiatric problems** | |
|  | The Parent Child Behavior Checklist Scores (abcd_cbcls01) were used to assess the dimensional psychopathology and adaptive functioning in children. CBCL has high test-retest stability and good internal consistency, and comprises of 113 items that measure broad scopes of child behavior across the past six months. Each item was rated using a three-point rating scale (not true, somewhat or sometimes true, very often or always true). It contains eight empirically-based syndrome scales related to psychiatric problems: anxious/depressed, withdrawn/depressed, somatic complaints, social problems, thought problems, attention problems, rule-breaking behavior, aggressive behavior. Internalizing and externalizing scores were derived from the following syndrome scores: anxious/depressed, withdrawn/depressed, somatic complaints, rule-breaking behavior and aggressive behavior. The total score of psychiatric problems is calculated by sum these sub-scores. The DSM-oriented scales have been introduced in the revision of CBCL after 2001. The DSM-oriented scales, including depressive problems (Depressive Problems CBCL DSM5 Scale), anxiety problems (Anxiety Problems CBCL DSM5 Scale), somatic problems (Somatic Problems CBCL DSM5 Scale), ADHD problems (ADHD CBCL DSM5 Scale), oppositional defiant problems (Oppositional Defiant Problems CBCL DSM5 Scale) and conduct problems (Conduct Problems CBCL DSM5 Scale), are based on the original CBCL item pool by expert ratings of similarity to DSM-IV criteria and have also been demonstrated to show good psychometric properties. In 2007, the CBCL officially identified 3 separate constructs including sluggish cognitive tempo (SCT), obsessive-compulsive problems (OCD) and stress, which has been used in previous studies. Therefore, there are 20 scores in the Parent Child Behavior Checklist Scores. |

**Table S3. Detailed description of registers used in the study**

| Data source | Information |
| --- | --- |
| National Patient Register | Provides information for all citizens on diagnoses and operations performed at a hospital since 1977. Each hospital discharge is recorded and classified according to the ICD-8 codes (1977-1993) and ICD-10 (1994 onwards) |
| National Prescription Register | Holds unique information on all redeemed prescriptions purchased by patients (medical treatment given only in hospital is not included) since 1995 |
| Danish Civil Registration System | Provides the unique individual personal identification number, information on sex, place of birth, marital status, and vital statistics since 1968 |
| The Danish Medical Birth Registry | The Medical Birth Registry includes birth characteristics, such as gestational age, birth weight, date of birth, sex, singleton or not since 1968 |
| The Danish Register of Cause of Death | Danish law mandates registration of the date of the cause of death for all Danish citizens who die in Denmark since 1970 |
| Integrated database for labour market research | Provides information on persons’ establishments and their relation since 1981 |
| Psychiatric central research register | Provides information of patients treated at psychiatric departments since 1969 |

**Table S4. The diagnostic classification of psychiatric disorders according to ICD-8 and ICD-10 system**

| Diagnosis | ICD-8 | | ICD-10 |
| --- | --- | --- | --- |
| Behavioral and emotional disorders with onset usually occurring in childhood and adolescence | | 306.x9, 308.0x | F90-F98 |
| Attention-deficit/hyperactivity disorders | | 308.01 | F90, F98.8 |
| Conduct disorders/oppositional defiant disorder | | 308.03, 308.04, 308.05, 308.06 | F91 |
| Emotional disorders | | 308.02 | F93 |
| Pervasive developmental disorders | | 299.00, 299.01, 299.02, 299.03 | F84 |
| Autism spectrum disorders | | 299.00 | F84.0 |
| Developmental disorders | | 306.0x, 306.1x, 306.3x | F80-83 |

**Table S5.** **Brain** **regions with their cortical volume or area significantly altered in the children whose mothers had SNVP during pregnancy** (FDR corrected, p<0.05).

| **Brain region** | **t value** | **Cohen’s d** | **p value** | **FDR p** |
| --- | --- | --- | --- | --- |
| Cortical volume in mm^3 for left hemisphere cortical Destrieux ROI total | -4.01 | -0.078 | 0.00006 | 0.011 |
| Cortical volume in mm^3 for right hemisphere cortical Destrieux ROI total | -3.98 | -0.077 | 0.00007 | 0.011 |
| Cortical volume in mm^3 for cortical Destrieux ROI total | -4.02 | -0.078 | 0.00006 | 0.011 |
| Cortical volume in mm^3 for left hemisphere cortical Destrieux ROI middle frontal gyrus | -3.70 | -0.072 | 0.00022 | 0.020 |
| Cortical volume in mm^3 for left hemisphere cortical Destrieux ROI postcentral gyrus | -3.69 | -0.071 | 0.00022 | 0.020 |
| Cortical area in mm^2 for left hemisphere cortical Destrieux ROI anterior part of the cingulate gyrus and sulcus | -3.30 | -0.064 | 0.00096 | 0.027 |
| Cortical area in mm^2 for left hemisphere cortical Destrieux ROI lateral occipito-temporal gyrus | -3.39 | -0.066 | 0.00070 | 0.027 |
| Cortical area in mm^2 for left hemisphere cortical Destrieux ROI central sulcus | -3.32 | -0.064 | 0.00090 | 0.027 |
| Cortical area in mm^2 for right hemisphere cortical Destrieux ROI anterior part of the cingulate gyrus and sulcus | -3.42 | -0.066 | 0.00064 | 0.027 |
| Cortical area in mm^2 for right hemisphere cortical Destrieux ROI precuneus | -3.34 | -0.065 | 0.00084 | 0.027 |
| Cortical area in mm^2 for left hemisphere cortical Destrieux ROI total | -3.55 | -0.069 | 0.00039 | 0.027 |
| Cortical area in mm^2 for right hemisphere cortical Destrieux ROI total | -3.43 | -0.066 | 0.00060 | 0.027 |
| Cortical area in mm^2 for cortical Destrieux ROI total | -3.51 | -0.068 | 0.00044 | 0.027 |
| Cortical volume in mm^3 for left hemisphere cortical Destrieux ROI superior frontal gyrus | -3.37 | -0.065 | 0.00076 | 0.027 |
| Cortical volume in mm^3 for left hemisphere cortical Destrieux ROI temporal pole | -3.35 | -0.065 | 0.00081 | 0.027 |
| Cortical volume in mm^3 for right hemisphere cortical Destrieux ROI superior parietal lobule | -3.43 | -0.066 | 0.00060 | 0.027 |
| Cortical area in mm^2 for left hemisphere cortical Destrieux ROI superior segment of the circular sulcus of the insula | -3.20 | -0.062 | 0.00137 | 0.033 |
| Cortical volume in mm^3 for left hemisphere cortical Destrieux ROI lateral occipito-temporal gyrus | -3.22 | -0.062 | 0.00130 | 0.033 |
| Cortical volume in mm^3 for right hemisphere cortical Destrieux ROI precuneus | -3.23 | -0.062 | 0.00126 | 0.033 |
| Cortical area in mm^2 for left hemisphere cortical Destrieux ROI transverse frontopolar gyri and sulci | -3.13 | -0.061 | 0.00174 | 0.038 |
| Cortical area in mm^2 for left hemisphere cortical Destrieux ROI middle frontal gyrus | -3.12 | -0.060 | 0.00180 | 0.038 |
| Cortical volume in mm^3 for left hemisphere cortical Destrieux ROI transverse frontopolar gyri and sulci | -3.11 | -0.060 | 0.00187 | 0.038 |
| Cortical volume in mm^3 for left hemisphere cortical Destrieux ROI superior segment of the circular sulcus of the insula | -3.09 | -0.060 | 0.00204 | 0.040 |
| Cortical area in mm^2 for left hemisphere cortical Destrieux ROI postcentral gyrus | -3.05 | -0.059 | 0.00229 | 0.041 |
| Cortical volume in mm^3 for left hemisphere cortical Destrieux ROI anterior part of the cingulate gyrus and sulcus | -3.07 | -0.059 | 0.00217 | 0.041 |
| Cortical volume in mm^3 for right hemisphere cortical Destrieux ROI calcarine sulcus | -3.04 | -0.059 | 0.00234 | 0.041 |
| Cortical volume in mm^3 for right hemisphere cortical Destrieux ROI superior frontal gyrus | -3.03 | -0.059 | 0.00243 | 0.041 |
| Cortical volume in mm^3 for right hemisphere cortical Destrieux ROI intraparietal sulcus and transverse parietal sulci | -3.02 | -0.058 | 0.00258 | 0.042 |
| Cortical area in mm^2 for left hemisphere cortical Destrieux ROI superior frontal gyrus | -2.93 | -0.057 | 0.00341 | 0.046 |
| Cortical area in mm^2 for left hemisphere cortical Destrieux ROI precuneus | -2.89 | -0.056 | 0.00387 | 0.046 |
| Cortical area in mm^2 for left hemisphere cortical Destrieux ROI inferior temporal gyrus | -2.85 | -0.055 | 0.00440 | 0.046 |
| Cortical area in mm^2 for left hemisphere cortical Destrieux ROI postcentral sulcus | -2.90 | -0.056 | 0.00369 | 0.046 |
| Cortical area in mm^2 for right hemisphere cortical Destrieux ROI superior parietal lobule | -2.90 | -0.056 | 0.00372 | 0.046 |
| Cortical area in mm^2 for right hemisphere cortical Destrieux ROI superior segment of the circular sulcus of the insula | -2.85 | -0.055 | 0.00435 | 0.046 |
| Cortical area in mm^2 for right hemisphere cortical Destrieux ROI pericallosal sulcus | -2.90 | -0.056 | 0.00377 | 0.046 |
| Cortical volume in mm^3 for left hemisphere cortical Destrieux ROI precentral gyrus | -2.87 | -0.055 | 0.00412 | 0.046 |
| Cortical volume in mm^3 for left hemisphere cortical Destrieux ROI central sulcus | -2.95 | -0.057 | 0.00314 | 0.046 |
| Cortical volume in mm^3 for left hemisphere cortical Destrieux ROI postcentral sulcus | -2.85 | -0.055 | 0.00438 | 0.046 |
| Cortical volume in mm^3 for right hemisphere cortical Destrieux ROI anterior part of the cingulate gyrus and sulcus | -2.94 | -0.057 | 0.00327 | 0.046 |
| Cortical volume in mm^3 for right hemisphere cortical Destrieux ROI posterior-dorsal part of the cingulate gyrus | -2.92 | -0.056 | 0.00354 | 0.046 |
| Cortical volume in mm^3 for right hemisphere cortical Destrieux ROI triangular part of the inferior frontal gyrus | -2.86 | -0.055 | 0.00427 | 0.046 |
| Cortical volume in mm^3 for right hemisphere cortical Destrieux ROI postcentral gyrus | -2.89 | -0.056 | 0.00382 | 0.046 |
| Cortical volume in mm^3 for right hemisphere cortical Destrieux ROI superior segment of the circular sulcus of the insula | -2.89 | -0.056 | 0.00389 | 0.046 |

**Table S6. The mediations on psychiatric problems implemented in the children by** **the volume and area of different cortical regions in the effects of** **exposure to severe and prolonged nausea and vomiting in pregnancy (SNVP)** (FDR corrected, p<0.05).

| **Cortical volume** | | | | | | | | | | |
| --- | --- | --- | --- | --- | --- | --- | --- | --- | --- | --- |
| **Brain region** | **path A** | | **path B** | | **path C'** | | **path C** | | **path AB** | |
|  | **beta** | **p value** | **beta** | **p value** | **beta** | **p value** | **beta** | **p value** | **beta** | **p value** |
| left hemisphere anterior part of the cingulate gyrus and sulcus' | -0.092 | 4.94E-04 | -0.792 | 6.55E-06 | 4.75 | <1.0E-10 | 4.82 | <1.0E-10 | 0.073 | 6.62E-03 |
| left hemisphere middle frontal gyrus | -0.096 | 2.48E-04 | -0.846 | 1.80E-06 | 4.74 | <1.0E-10 | 4.82 | <1.0E-10 | 0.081 | 4.13E-03 |
| left hemisphere superior frontal gyrus | -0.088 | 5.62E-04 | -0.925 | 3.18E-07 | 4.74 | <1.0E-10 | 4.82 | <1.0E-10 | 0.082 | 4.76E-03 |
| left hemisphere lateral occipito-temporal gyrus | -0.089 | 9.44E-04 | -0.658 | 1.38E-04 | 4.77 | <1.0E-10 | 4.82 | <1.0E-10 | 0.058 | 1.43E-02 |
| left hemisphere postcentral gyrus | -0.105 | 7.87E-05 | -0.938 | 8.13E-08 | 4.73 | <1.0E-10 | 4.82 | <1.0E-10 | 0.098 | 1.66E-03 |
| left hemisphere precentral gyrus | -0.071 | 4.70E-03 | -0.823 | 7.21E-06 | 4.77 | <1.0E-10 | 4.82 | <1.0E-10 | 0.059 | 1.87E-02 |
| left hemisphere temporal pole | -0.087 | 9.48E-04 | -0.928 | 1.53E-07 | 4.74 | <1.0E-10 | 4.82 | <1.0E-10 | 0.080 | 5.74E-03 |
| left hemisphere central sulcus | -0.074 | 4.93E-03 | -0.881 | 5.64E-07 | 4.76 | <1.0E-10 | 4.82 | <1.0E-10 | 0.065 | 1.57E-02 |
| left hemisphere superior segment of the circular sulcus of the insula | -0.077 | 2.77E-03 | -1.029 | 9.63E-09 | 4.74 | <1.0E-10 | 4.82 | <1.0E-10 | 0.079 | 8.72E-03 |
| left hemisphere postcentral sulcus | -0.071 | 7.43E-03 | -0.634 | 2.79E-04 | 4.78 | <1.0E-10 | 4.82 | <1.0E-10 | 0.045 | 3.53E-02 |
| right hemisphere anterior part of the cingulate gyrus and sulcus | -0.088 | 7.46E-04 | -0.656 | 2.12E-04 | 4.77 | <1.0E-10 | 4.82 | <1.0E-10 | 0.058 | 1.45E-02 |
| right hemisphere superior frontal gyrus | -0.078 | 2.55E-03 | -1.099 | 1.01E-09 | 4.74 | <1.0E-10 | 4.82 | <1.0E-10 | 0.085 | 7.44E-03 |
| right hemisphere superior parietal lobule | -0.089 | 8.48E-04 | -0.685 | 8.53E-05 | 4.76 | <1.0E-10 | 4.82 | <1.0E-10 | 0.061 | 1.25E-02 |
| right hemisphere postcentral gyrus | -0.081 | 2.69E-03 | -0.784 | 4.79E-06 | 4.76 | <1.0E-10 | 4.82 | <1.0E-10 | 0.064 | 1.36E-02 |
| right hemisphere precuneus | -0.080 | 2.08E-03 | -0.815 | 4.43E-06 | 4.76 | <1.0E-10 | 4.82 | <1.0E-10 | 0.065 | 1.19E-02 |
| right hemisphere superior segment of the circular sulcus of the insula | -0.073 | 5.46E-03 | -0.650 | 2.17E-04 | 4.78 | <1.0E-10 | 4.82 | <1.0E-10 | 0.048 | 2.99E-02 |
| right hemisphere intraparietal sulcus and transverse parietal sulci | -0.076 | 4.52E-03 | -0.569 | 1.01E-03 | 4.78 | <1.0E-10 | 4.82 | <1.0E-10 | 0.043 | 3.62E-02 |
| **Cortical area** | | | | | | | | | | |
| left hemisphere anterior part of the cingulate gyrus and sulcus | -0.097 | 2.16E-04 | -0.854 | 1.34E-06 | 4.74 | <1.0E-10 | 4.82 | <1.0E-10 | 0.083 | 3.74E-03 |
| left hemisphere middle frontal gyrus | -0.081 | 1.66E-03 | -0.991 | 3.79E-08 | 4.74 | <1.0E-10 | 4.82 | <1.0E-10 | 0.080 | 6.98E-03 |
| left hemisphere superior frontal gyrus | -0.076 | 2.40E-03 | -1.137 | 9.32E-10 | 4.74 | <1.0E-10 | 4.82 | <1.0E-10 | 0.086 | 7.11E-03 |
| left hemisphere lateral occipito-temporal gyrus | -0.097 | 3.83E-04 | -0.683 | 5.82E-05 | 4.76 | <1.0E-10 | 4.82 | <1.0E-10 | 0.066 | 8.87E-03 |
| left hemisphere postcentral gyrus | -0.080 | 2.02E-03 | -1.030 | 8.38E-09 | 4.74 | <1.0E-10 | 4.82 | <1.0E-10 | 0.082 | 7.14E-03 |
| left hemisphere precuneus | -0.077 | 3.62E-03 | -0.711 | 4.79E-05 | 4.77 | <1.0E-10 | 4.82 | <1.0E-10 | 0.055 | 2.03E-02 |
| left hemisphere inferior temporal gyrus | -0.073 | 4.94E-03 | -0.650 | 2.59E-04 | 4.78 | <1.0E-10 | 4.82 | <1.0E-10 | 0.048 | 2.94E-02 |
| left hemisphere central sulcus | -0.082 | 1.18E-03 | -1.000 | 5.46E-08 | 4.74 | <1.0E-10 | 4.82 | <1.0E-10 | 0.082 | 5.92E-03 |
| left hemisphere superior segment of the circular sulcus of the insula | -0.082 | 1.38E-03 | -1.121 | 4.65E-10 | 4.73 | <1.0E-10 | 4.82 | <1.0E-10 | 0.092 | 4.84E-03 |
| left hemisphere postcentral sulcus | -0.070 | 6.95E-03 | -0.575 | 1.21E-03 | 4.78 | <1.0E-10 | 4.82 | <1.0E-10 | 0.040 | 4.37E-02 |
| right hemisphere anterior part of the cingulate gyrus and sulcus | -0.097 | 1.74E-04 | -0.842 | 2.42E-06 | 4.74 | <1.0E-10 | 4.82 | <1.0E-10 | 0.082 | 3.76E-03 |
| right hemisphere superior parietal lobule | -0.074 | 5.18E-03 | -0.579 | 1.01E-03 | 4.78 | <1.0E-10 | 4.82 | <1.0E-10 | 0.043 | 3.80E-02 |
| right hemisphere precuneus | -0.086 | 1.08E-03 | -0.675 | 1.28E-04 | 4.77 | <1.0E-10 | 4.82 | <1.0E-10 | 0.058 | 1.47E-02 |
| right hemisphere superior segment of the circular sulcus of the insula | -0.072 | 5.79E-03 | -0.746 | 2.50E-05 | 4.77 | <1.0E-10 | 4.82 | <1.0E-10 | 0.054 | 2.35E-02 |
| right hemisphere pericallosal sulcus | -0.078 | 3.45E-03 | -0.596 | 5.80E-04 | 4.78 | <1.0E-10 | 4.82 | <1.0E-10 | 0.047 | 2.96E-02 |

**Table S7. The mediations implemented in the children by the volume and area of different cortical regions in the effects of exposure to SNVP on cognition** (FDR corrected, p<0.05)**.**

| **Cortical volume** | | | | | | | | | | |
| --- | --- | --- | --- | --- | --- | --- | --- | --- | --- | --- |
| **Brain region** | **path A** | | **path B** | | **path C'** | | **path C** | | **path AB** | |
|  | **beta** | **p value** | **beta** | **p value** | **beta** | **p value** | **beta** | **p value** | **beta** | **p value** |
| left hemisphere transverse frontopolar gyri and sulci | -0.086 | 1.42E-03 | 0.243 | 1.63E-03 | -0.89 | 2.87E-05 | -0.92 | 1.86E-05 | -0.021 | 2.87E-02 |
| left hemisphere anterior part of the cingulate gyrus and sulcus | -0.096 | 2.98E-04 | 0.676 | <1.0E-10 | -0.85 | 6.63E-05 | -0.92 | 1.86E-05 | -0.065 | 9.08E-04 |
| left hemisphere middle frontal gyrus | -0.105 | 6.90E-05 | 0.496 | 3.71E-10 | -0.86 | 5.28E-05 | -0.92 | 1.86E-05 | -0.052 | 8.68E-04 |
| left hemisphere superior frontal gyrus | -0.091 | 4.03E-04 | 0.506 | 3.95E-10 | -0.87 | 4.69E-05 | -0.92 | 1.86E-05 | -0.046 | 2.28E-03 |
| left hemisphere lateral occipito-temporal gyrus | -0.094 | 5.42E-04 | 0.425 | 3.40E-08 | -0.88 | 4.15E-05 | -0.92 | 1.86E-05 | -0.040 | 3.76E-03 |
| left hemisphere postcentral gyrus | -0.107 | 6.18E-05 | 0.407 | 1.92E-07 | -0.87 | 4.50E-05 | -0.92 | 1.86E-05 | -0.044 | 1.69E-03 |
| left hemisphere precentral gyrus | -0.077 | 2.58E-03 | 0.647 | 2.89E-15 | -0.87 | 4.91E-05 | -0.92 | 1.86E-05 | -0.050 | 5.17E-03 |
| left hemisphere temporal pole | -0.090 | 6.50E-04 | 0.616 | 5.77E-15 | -0.86 | 5.55E-05 | -0.92 | 1.86E-05 | -0.056 | 1.91E-03 |
| left hemisphere central sulcus | -0.078 | 3.12E-03 | 0.551 | 2.77E-12 | -0.87 | 4.36E-05 | -0.92 | 1.86E-05 | -0.043 | 6.94E-03 |
| left hemisphere superior segment of the circular sulcus of the insula | -0.082 | 1.56E-03 | 0.665 | <1.0E-10 | -0.86 | 5.42E-05 | -0.92 | 1.86E-05 | -0.055 | 3.29E-03 |
| left hemisphere postcentral sulcus | -0.070 | 9.03E-03 | 0.391 | 5.08E-07 | -0.89 | 3.22E-05 | -0.92 | 1.86E-05 | -0.027 | 2.25E-02 |
| right hemisphere anterior part of the cingulate gyrus and sulcus | -0.092 | 4.76E-04 | 0.661 | <1.0E-10 | -0.85 | 6.14E-05 | -0.92 | 1.86E-05 | -0.061 | 1.35E-03 |
| right hemisphere posterior-dorsal part of the cingulate gyrus | -0.084 | 2.10E-03 | 0.508 | 2.85E-11 | -0.87 | 4.34E-05 | -0.92 | 1.86E-05 | -0.043 | 5.66E-03 |
| right hemisphere superior frontal gyrus | -0.085 | 1.04E-03 | 0.571 | 1.32E-12 | -0.87 | 4.86E-05 | -0.92 | 1.86E-05 | -0.049 | 3.14E-03 |
| right hemisphere superior parietal lobule | -0.089 | 9.11E-04 | 0.348 | 8.00E-06 | -0.88 | 3.49E-05 | -0.92 | 1.86E-05 | -0.031 | 8.77E-03 |
| right hemisphere postcentral gyrus | -0.083 | 2.39E-03 | 0.306 | 6.51E-05 | -0.89 | 3.12E-05 | -0.92 | 1.86E-05 | -0.025 | 1.77E-02 |
| right hemisphere precuneus | -0.083 | 1.69E-03 | 0.389 | 9.69E-07 | -0.88 | 3.56E-05 | -0.92 | 1.86E-05 | -0.032 | 9.17E-03 |
| right hemisphere calcarine sulcus | -0.081 | 2.62E-03 | 0.405 | 1.66E-07 | -0.88 | 3.61E-05 | -0.92 | 1.86E-05 | -0.033 | 1.00E-02 |
| right hemisphere superior segment of the circular sulcus of the insula | -0.080 | 2.63E-03 | 0.453 | 8.40E-09 | -0.88 | 3.84E-05 | -0.92 | 1.86E-05 | -0.036 | 8.40E-03 |
| right hemisphere intraparietal sulcus and transverse parietal sulci | -0.075 | 5.56E-03 | 0.471 | 1.07E-09 | -0.88 | 3.75E-05 | -0.92 | 1.86E-05 | -0.035 | 1.25E-02 |
| **Cortical area** | | | | | | | | | | |
| left hemisphere transverse frontopolar gyri and sulci | -0.090 | 1.06E-03 | 0.421 | 2.74E-08 | -0.88 | 3.99E-05 | -0.92 | 1.86E-05 | -0.038 | 5.30E-03 |
| left hemisphere anterior part of the cingulate gyrus and sulcus | -0.103 | 9.33E-05 | 0.856 | <1.0E-10 | -0.83 | 1.02E-04 | -0.92 | 1.86E-05 | -0.088 | 2.48E-04 |
| left hemisphere middle frontal gyrus | -0.090 | 5.09E-04 | 0.700 | <1.0E-10 | -0.85 | 6.36E-05 | -0.92 | 1.86E-05 | -0.063 | 1.33E-03 |
| left hemisphere superior frontal gyrus | -0.081 | 1.30E-03 | 0.713 | <1.0E-10 | -0.86 | 5.72E-05 | -0.92 | 1.86E-05 | -0.058 | 2.74E-03 |
| left hemisphere lateral occipito-temporal gyrus | -0.105 | 1.26E-04 | 0.482 | 2.12E-10 | -0.86 | 5.15E-05 | -0.92 | 1.86E-05 | -0.051 | 1.14E-03 |
| left hemisphere postcentral gyrus | -0.083 | 1.50E-03 | 0.493 | 7.12E-10 | -0.87 | 4.20E-05 | -0.92 | 1.86E-05 | -0.041 | 5.21E-03 |
| left hemisphere precuneus | -0.085 | 1.43E-03 | 0.404 | 2.39E-07 | -0.88 | 3.73E-05 | -0.92 | 1.86E-05 | -0.034 | 7.40E-03 |
| left hemisphere inferior temporal gyrus | -0.078 | 2.94E-03 | 0.598 | 5.26E-14 | -0.87 | 4.65E-05 | -0.92 | 1.86E-05 | -0.047 | 6.05E-03 |
| left hemisphere central sulcus | -0.087 | 5.84E-04 | 0.669 | 4.44E-16 | -0.86 | 5.84E-05 | -0.92 | 1.86E-05 | -0.058 | 1.64E-03 |
| left hemisphere superior segment of the circular sulcus of the insula | -0.090 | 5.30E-04 | 0.787 | <1.0E-10 | -0.84 | 7.29E-05 | -0.92 | 1.86E-05 | -0.071 | 1.14E-03 |
| left hemisphere postcentral sulcus | -0.070 | 8.04E-03 | 0.465 | 4.69E-09 | -0.88 | 3.55E-05 | -0.92 | 1.86E-05 | -0.032 | 1.70E-02 |
| right hemisphere anterior part of the cingulate gyrus and sulcus | -0.104 | 7.05E-05 | 0.834 | <1.0E-10 | -0.83 | 9.88E-05 | -0.92 | 1.86E-05 | -0.087 | 2.14E-04 |
| right hemisphere superior parietal lobule | -0.075 | 4.99E-03 | 0.436 | 2.89E-08 | -0.88 | 3.57E-05 | -0.92 | 1.86E-05 | -0.032 | 1.34E-02 |
| right hemisphere precuneus | -0.090 | 7.20E-04 | 0.421 | 9.15E-08 | -0.88 | 3.98E-05 | -0.92 | 1.86E-05 | -0.038 | 4.76E-03 |
| right hemisphere superior segment of the circular sulcus of the insula | -0.080 | 2.30E-03 | 0.553 | 2.59E-12 | -0.87 | 4.48E-05 | -0.92 | 1.86E-05 | -0.044 | 5.57E-03 |
| right hemisphere pericallosal sulcus | -0.084 | 1.86E-03 | 0.483 | 4.37E-10 | -0.88 | 4.17E-05 | -0.92 | 1.86E-05 | -0.041 | 5.83E-03 |

**Table S8 Incidence rate and hazard ratio of specific psychiatric disorders** in offspring born during 1995-2012 in Denmark according to maternal hyperemesis gravidarum

|  | No of Cases | Incidence rate | Model 1 | Model 2 |
| --- | --- | --- | --- | --- |
|  | N | Rate per 1000 person-years | HR (95% CI) | HR (95% CI) |
| Behavioral and emotional disorders^*^ | | | | |
| No HG | 41 869 | 3.07 | REF | REF |
| Maternal HG | 595 | 3.67 | 1.27 (1.17-1.38) | 1.20 (1.10-1.30) |
| Attention-deficit/hyperactivity disorders | | | | |
| No HG | 32 177 | 2.57 | REF | REF |
| Maternal HG | 437 | 2.85 | 1.23 (1.12-1.35) | 1.16 (1.06-1.28) |
| Conduct disorders/oppositional defiant disorders | | | | |
| No HG | 2024 | 0.15 | REF | REF |
| Maternal HG | 29 | 0.18 | 1.31 (0.91-1.89) | 1.06 (1.71-1.57) |
| Emotional disorders | | | | |
| No HG | 2884 | 0.21 | REF | REF |
| Maternal HG | 44 | 0.27 | 1.34 (1.00-1.81) | 1.33 (0.98-1.89) |
| Pervasive developmental disorders | | | | |
| No HG | 17 478 | 1.31 |  |  |
| Maternal HG | 232 | 1.46 | 1.23 (1.08-1.40) | 1.19 (1.05-1.36) |
| Childhood autism | | | | |
| No HG | 6877 | 0.50 | REF | REF |
| Maternal HG | 100 | 0.61 | 1.32 (1.08-1.61) | 1.19 (0.97-1.45) |
| Developmental disorders^#^ | | | | |
| No HG | 3362 | 0.24 | REF | REF |
| Maternal HG | 55 | 0.33 | 1.43 (1.10-1.86) | 1.33 (1.02-1.75) |

HG: hyperemesis gravidarum; HR: Hazard ratio; Model 1= children’s age at time scale; Model 2= children’s age at time scale, sex, birth of year, parity, parental age at birth, maternal education level, maternal country of origin, maternal cohabitation and parental psychiatry disorders; *indicates behavioral and emotional disorders with onset usually occurring in childhood and adolescence; #indicates developmental disorders including language, learning and motor skills disorders

Live-born singletons during 1978-2012 in Denmark (n= 2,100,158)

Excluded

1. Children with likely error in gestational age (n=463)
2. Children with missing links to their fathers (n=6798)

Included in the final analysis (n=2,092,897)

Unexposed children whose

mothers were without HG

(n=21 282)

Exposed children whose

mothers were with HG

(n=2 071 615)

**Figure S1 Flowchart showing the identification of the eligible participants and analysis sample**


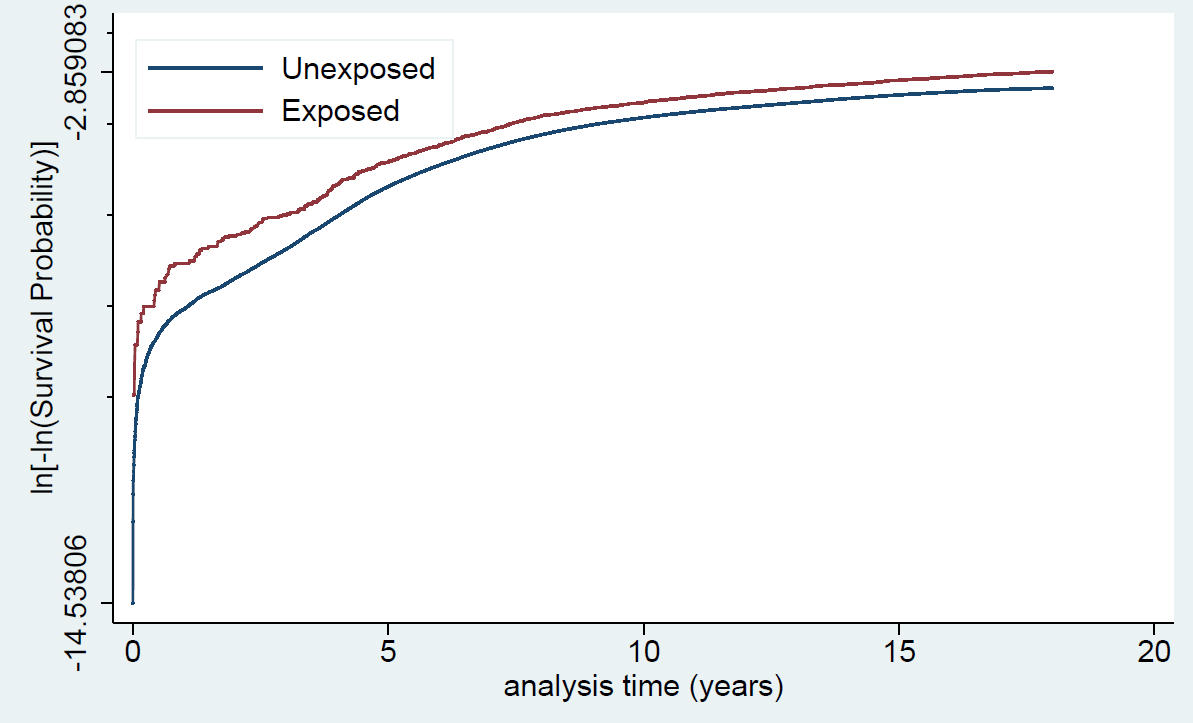


**Figure S2 The log-minus-log survival curve**

**Supplementary Figure 3 The proportion of offspring born to mothers with**

**hyperemesis gravidarum by birth year**
